# How many antiviral small interfering RNAs may be encoded by the mammalian genomes?

**DOI:** 10.1186/1745-6150-5-62

**Published:** 2010-11-08

**Authors:** Anastasia Zabolotneva, Victor Tkachev, Felix Filatov, Anton Buzdin

**Affiliations:** 1 Shemyakin-Ovchinnikov Institute of Bioorganic Chemistry, Russian Academy of Sciences, 16/10 Miklukho-Maklaya st, Moscow 117997, Russia; 2 Deutsche Bank Russia, Sadovnicheskaya 82 building 2, Moscow 117312, Russia; 3 Hematology Research Center, Russian Academy of medical sciences, 4a Novy Zykovsky Pr., Moscow 125167, Russia

## Abstract

**Background:**

The discovery of RNA interference phenomenon (RNAi) and understanding of its mechanisms has revolutionized our views on many molecular processes in the living cell. Among the other, RNAi is involved in silencing of transposable elements and in inhibition of virus infection in various eukaryotic organisms. Recent experimental studies demonstrate few cases of viral replication suppression via complementary interactions between the mammalian small RNAs and viral transcripts.

**Presentation of the hypothesis:**

It was found that >50% of the human genome is transcribed in different cell types and that these transcripts are mainly not associated with known protein coding genes, but represent non-coding RNAs of unknown functions. We propose a hypothesis that mammalian DNAs encode thousands RNA motifs that may serve for antiviral protection. We also presume that the evolutional success of some groups of genomic repeats and, in particular, of transposable elements (TEs) may be due to their ability to provide antiviral RNA motifs to the host organism. Intense genomic repeat propagation into the genome would inevitably cause bidirectional transcription of these sequences, and the resulting double-stranded RNAs may be recognized and processed by the RNA interference enzymatic machinery. Provided that these processed target motifs may be complementary to viral transcripts, fixation of the repeats into the host genome may be of a considerable benefit to the host. It fits with our bioinformatical data revealing thousands of 21-28 bp long motifs identical between human DNA and human-pathogenic adenoviral and herpesviral genomes. Many of these motifs are transcribed in human cells, and the transcribed part grows proportionally to their lengths. Many such motifs are included in human TEs. For example, one 23 nt-long motif that is a part of human abundant Alu retrotransposon, shares sequence identity with eight human adenoviral genomes.

**Testing the hypothesis:**

This hypothesis could be tested on various mammalian species and viruses infecting mammalian cells.

**Implications of the hypothesis:**

This hypothesis proposes that mammalian organisms may use their own genomes as sources of thousands of putative interfering RNA motifs that can be recruited to repress intracellular pathogens like proliferating viruses.

**Reviewers:**

This article was reviewed by Eugene V. Koonin, Valerian V. Dolja and Yuri V. Shpakovski.

## Background

The discovery of RNA interference (RNAi) and understanding of its mechanisms has opened a new era in molecular genetics. It is now clear that very small complementary RNAs may modulate expression of large genes. RNAi and related mechanisms may interfere with gene expression at the stages of transcription, processing of mRNA and translation [1,2]. They may alter transcript stability and may even cause methylation of extended genomic loci [3,4]. RNAi is a very conservative mechanism that is likely to be active in almost all eukaryotic taxa. This phenomenon attracts growing attention and studying RNA interference is probably one of the most rapidly developing fields of modern science. Known RNAi pathways are very diverse [2] and many new mechanisms are probably still to be discovered [5]. A fundamental step of RNAi is the basepairing of the two interacting RNAs. The resulting duplexes, that may be perfectly or not perfectly matched, are further recognized by the cellular RNAi machinery which may result in silencing of the source gene for one of the above interacting RNAs. The complementary motifs in RNA may be only 21 nucleotides long or even smaller [6,7]. At present, RNAi is known to control genes involved in all fields of cell functioning, including proliferation, growth, differentiation and cell death [1,8,9]. There are also some "exotic" functions like the control of genomic transposable elements. Transposable elements (TEs) are "selfish" fragments of genomic DNA able to self-reproduce and to insert into new locations into the host genome. TEs occupy huge space in eukaryotic DNA, e.g. they account for at least 50% of the human genome [10] and 50-90% of the genomes of many plant species [11]. Different TE families may be represented in genomes by a very different number of representatives like tens, hundreds, thousands, and even millions of TE copies per genome. Although TE copies fixed in the genome are most frequently neutral or even advantageous to the host organism, their uncontrolled proliferation and insertional activity may cause multiple genetic and developmental deleterious effects [12-16].

At least in several species, TE proliferation is controlled by the RNAi mechanisms [17,18]. For example, in the DNA of fruitfly *Drosophila melanogaster *there are several conserved loci that do not encode any functional genes, but instead contain mutated copies of some TEs [19]. These loci are transcribed in both sense and antisense orientations, which results in generation of large double-stranded RNAs (dsRNAs) including TE sequences. Such dsRNAs are recognized by the cellular RNAi machinery that further represses expression of all genomic TEs identical to those located in the above loci. When these loci are highly transcriptionally active, protection against TE expression is strong, when they are silent or hardly transcribed - the protection is weak [20,21].

## Presentation of the hypothesis

In the DNAs of some prokaryotes (*e.g., E. coli*), there are ~50 bp long sequence motifs identical to fragments of bacteriophagal genomes. It was shown that the presence of these motifs in the bacterial DNA protects them from bacteriophagal infection, although the mechanism of this protection (probably not RNAi) remains a mystery [22]. Also, it was recently hypothesized that there may be a specific mechanism in *crustaceans *that provides reverse transcription of viral transcripts and subsequent insertion of the resulting cDNAs into the genome. Further transcription of these cDNAs in the antisense orientation may help the host organisms to resist future viral infections by acting as an intracellular specific immunity system [23]. There is a growing number of instances of acquisition of RNA virus sequences by eukaryotic genomes that were proposed to function in antiviral defense [24]. However, at present, the mechanism of the RNAi based antiviral protection in eukaryotic organisms has not been sufficiently investigated [25]. Theoretically, RNAi could well serve as intracellular "immune system" by repressing transcription of not only intra-genomic parasites like TEs, but also of external ones like viruses. In plants and in invertebrates, many cases of viral gene suppression using small interfering RNAs originating from viral double stranded RNAs have been documented to the date [26]. Furthermore, several examples of virus-encoded small interfering RNAs that may regulate host gene expression became available recently [27]. Finally, at least in the four cases mammalian siRNAs are thought to interfere with viral transcripts, thus preventing efficient virus replication [28-31].

### Eukaryotic genomes differ greatly in size

Genome sizes in eukaryotes may vary more than 50.000-fold, from ~ 12 × 10^6 ^bp like in the case of yeast *Saccharomyces cerevisiae *haploid genome [32], and up to over 670 × 10^9 ^bp, like for protist *Amoeba dubia *[33]. In vertebrate organisms, sizes differ from ~ 380 × 10^6 ^bp (puffer fish *Tetraodon nigroviridis*) to ~130 × 10^9 ^bp (marbled lungfish *Protopterus aethiopicus*) with the intermediate value of ~3 × 10^9 ^for human haploid genome [33].

The genome growth occurs by virtue of various processes the most important of which are random DNA duplication and propagation of transposable elements [34,35]. In the large eukaryotic DNAs, protein coding sequences occupy a rather modest fraction (a few percent or lower), whereas significantly bigger parts of these genomes appear to be transcribed. For example, according to the published data, more than 50% of the human DNA is transcribed in different cell types [36]. These transcripts are frequently not associated with any known genes, but represent non-coding RNAs of unknown functions [37]. At least part of these transcripts is likely to participate in RNAi-mediated regulation of gene expression.

### The hypothesis

We propose a hypothesis that virus suppression mediated by self-encoded small interfering RNAs is not an exception but is rather a general case for the mammalian genomes and, probably, for other relatively big eukaryotic genomes. These genomes may include relatively short motifs sharing high sequence identity with viral genes, and, when transcribed, these motifs may function for the antiviral host cell defense. In this light the enlargement of genome sizes may be beneficial to the host organisms as a source of novel putative interfering RNA motifs that can be recruited to repress intracellular pathogens like proliferating viruses. An increased genome would allow an "empty space" for the evolution of different genetic elements, including non-coding DNA. Casual combinations of nucleotides in the new part of the genome might create new DNA motifs that theoretically, after being transcribed, could be used by the host organism as a tool for recognition and targeting of intracellular pathogen transcripts. Novel transcribed DNA motifs that would target the host genes would be eliminated from the genome, whereas those that complementarily match with the pathogen RNAs would be positively selected. Neutral motifs could be "stored" in the genomes as ordinary non-coding DNA.

Mechanisms of genome size increase might include DNA duplications, expansion of satellite sequences, emergence of polyploid chromosomes, and insertions of transposable elements [38]. Initially, the newly amplified part of the genome is identical or very close to the "progenitor" genomic DNA. However, neutral or non-neutral mutation pressure may form a novel DNA landscape within the amplified fragments during genome evolution. In case of being transcribed, these loci might significantly increase the repertoire of cellular interfering RNAs.

We also presume that the evolutional success of some groups of genomic repeats and, in particular, transposable elements (TEs) at least partly may be due to their ability to provide antiviral RNA motifs to the host organism. Intense propagation of the repetitive elements into the genome would necessarily cause bidirectional transcription of these sequences, and the resulting double-stranded RNAs may be recognized and processed by the RNA interference enzymatic machinery. Provided that these processed target motifs may be complementary to viral transcripts, fixation of the repeats into the host genome may be of a considerable benefit to the host.

**Figure 1:**
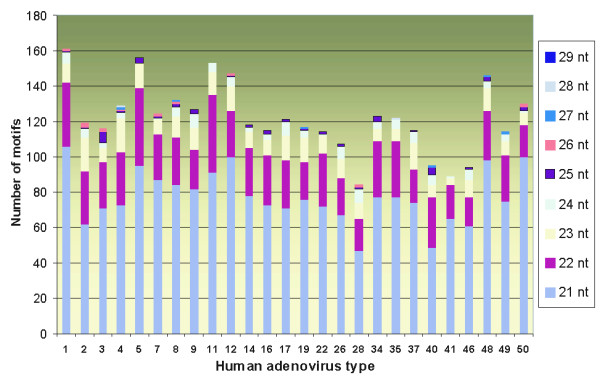
**Human genome contains sequence motifs identical to the DNAs of human adenoviruses**. Human adenovirus types are shown on abscises axis. Histogram represents numbers of 21-, 22-, 23-, 24-, 25-, 26-, 27-, 28- and 29 nucleotide long sequence motifs identical between human and adenoviral genomes. Complete human adenovirus genome sequences were extracted from GenBank. Quantization of perfectly matching nucleotide motifs was done using the BLAST Web-server at NCBI [44].

We performed a bioinformatic assay aimed to quantify in human DNA sequence motifs that perfectly match on 26 published adenoviral genomes (Figure 1). Human adenoviral genomes have similar lengths of ~34-36 kb and encode each for approximately 35 viral genes [39]. Only different nucleotide motifs were taken into account, motifs repeated in human or adenoviral genome several times were considered as a sole motif. Each motif was quantified only once, *e.g*. 25 nt-long sequence was registered only as one 25 nt-long motif, but not also as 24-, 23-, 22- and 21 nt-long motifs.

For different human adenoviruses, we identified 47-106 perfectly matched *21 *nt-long motifs, 16-44 *22 *nt-long, 4-19 *23 *nt-long, 0-8 *24 *nt-long, 0-6 *25 *nt-long, 0-6 *26 *nt-long and 0-1 *27-*, *28- *and *29 *nt-long motifs per genome. The overall number of such motifs varied from 85 to 161. Provided that more than 50% of human DNA is transcribed, and that this transcription may be driven in both directions, we may expect that more than a quarter of the above complementary motifs are transcribed within the RNA molecules in the antisense orientation relatively to adenoviral gene transcriptional direction. At least theoretically these motifs might be somehow involved in downregulation of viral genes.

**Figure 2:**
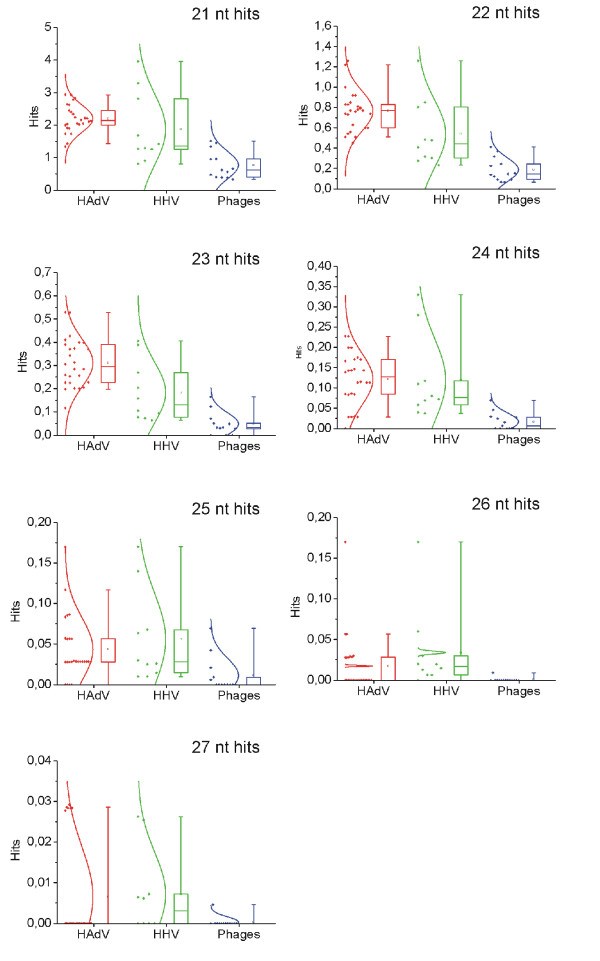
**Relative content of sequences identical to human DNA in human adenoviral (HAdV), human herpesviral (HHV) and bacteriophagal genomes**. Ordinate axis represents the average number of BLAST hits per 1 kb of virus genome sequence calculated for each individual virus under investigation. Results for adenoviruses are shown in red, for herpes viruses - in green and for phages - in blue. Probability density functions are shown for normal distribution model. Bars delineate, where applicable, percentiles for 5, 25, 50, 75 and 95% probabilities. Empty boxes represent average values. Graphs were built using OriginPro 8 software.

Similar data were obtained when comparing human DNA with 10 human pathogenic herpesvirus genomes. We further compared relative occurrences of 21-29 nt long hits among adenoviral, herpesviral and bacteriophagal genomes (a list of the investigated viral genomes can be found in Table 1). To this end, the number of the respective identified BLAST hits was normalized to 1 kb of each virus genome sequence. The resulting figure clearly shows an approximately 3-fold greater average number of hits for adenoviral and herpesviral genomes rather than for bacteriophages in all size ranges (Figure 2). The excess of hits in human-pathogenic virus genomes compared to bacteriophages was statistically significant with p-values < 0,01 for 21-25 nt-long hits (p-values shown on Table 1).

**Table 1:** P-values calculated for the distribution of perfectly matching human DNA 21-27 nt-long hits between the different types of viral and randomly generated genomes.

**Hits, nt-long**^**1**^	**AV-Phage**^**2**^	**HV-Phage**^**3**^	**HV-AV**^**4**^
21	<0,0001	0,003	0,19

22	<0,0001	0,0017	0,016

23	<0,0001	0,0023	0,004

24	<0,0001	0,0019	0,94

25	0,0022	0,0032	0,99

26	0,013	0,0005	0,06

27	0,38	0,058	0,48

^1 ^P-values were calculated using GraphPadPrism software. For 21-24 nt-long hits, t-test for normal distribution was used; for 25-27 nt-long hits, non-parametric Mann-Whitney test was used.

^2^P-values for the comparison of adenoviral and bacteriophagal hits.

^3^P-values for the comparison of herpesviral and bacteriophagal hits.

^4^P-values for the comparison of adenoviral and herpesviral hits.

Alternatively, we compared human-virus sequence identities using a panel of randomly generated genomes. Using 2^nd ^- and 5^th ^order Markov model we generated random sequences by shuffling the actual adenoviral, herpesviral and bacteriophagal genomes. Under this approach, 1000 random sequences were generated separately for each investigated viral genome. We next compared numbers of BLAST hits for the existing viral genomes, and for *in silico *generated ones. The observed BLAST hits (total numbers of BLAST hits were found for each genome) were statistically analyzed (Table 2), and the following was found: (*i*) the number of hits for the existing adenoviral or herpesviral genome was mostly higher than the 95^th ^percentile for a set of the corresponding *in silico*-generated sequences (in 81% or 89% of the cases, respectively, for 5^th ^order Markov model), (*ii*) for the bacteriophages, this number was mostly below the 95% percentile and expanded it only in 38% of the cases). Again, these data confirm that human-pathogenic viruses share significantly greater structural identity with the human genome than do the bacteriophagal genomes. Importantly, this also implies that there was a kind of positive selection for either the "virus-like" sequences in the human DNA, or for the "humanized" DNA in the human-pathogenic viruses, or both.

**Table 2:** BLAST hits found for the existing and randomly generated genomes.

**Virus**^**1**^	Accession number (NCBI PubMed)	**Original Genome**^**2**^	**Markov 2**^**nd **^**order, percentile**^**3**^			**Markov 5**^**th **^**order, percentile**^**3**^		
			
			5	50	95	5	50	95
av-01	[GenBank:AC_000017] [GenBank:BK005234]	**368**	10	20	36,95	15	30	56
av-02	[GenBank:AC_000007]	**1413**	10	20	35	14,05	29	54,95
av-03	[GenBank:AY599834]	**561**	14	28	47	22	39	62,95
av-04	[GenBank:AY599837]	**70**	9,05	19	42,9	16	28	60,95
av-05	[GenBank:AC_000008]	**92**	9,05	20	37	14	28	63,95
av-06	[GenBank:FJ349096]	**55**	19	35	58	24,05	47	126,9
av-07	[GenBank:AC_000018] [GenBank:BK005235]	**498**	16	27	46,95	23,05	41	76,85
av-08	[GenBank:AB448769]	**171**	11	22	40	16	32	56
av-09	[GenBank:AJ854486]	**113**	8	18	28,95	14,05	26	50
av-10-02	[GenBank:AB023548]	**821**	9	21	43	18	32	64
av-10-03	[GenBank:AB330091]	**55**	23	47	68			
av-10	[GenBank:DQ149615]	**84**	61	76	165,7	62	81	186,9
av-11	[GenBank:AC_000015] [GenBank:BK001453]	**101**	18	30	54,95	27,05	45	88,85
av-12	[GenBank:X73487]	**132**	24	42	68,9	33	52	80
av-13-02	[GenBank:DQ149616]	**72**	19,05	47	73,95			
av-13	[GenBank:AB330094]	**815**	10	22	44,95	18	33	79,95
av-14	[GenBank:FJ822614]	**63**	18	31	53,95	25	42	72,9
av-15	[GenBank:DQ149617]	**814**	11,05	23	46,8	15	31	62
av-16	[GenBank:AY601636]	**75**	13,05	27	47,95	21	38	66
av-17	[GenBank:AF108105]	**89**	8	18,5	34,9	14	27	51
av-18	[GenBank:GU191019]	**45**	19	33	67,95	20	36	67,95
av-19	[GenBank:AB448774]	**162**	9,05	19	36	14	27	48
av-20	[GenBank:DQ149619]	**813**	12	25	49,95			
av-21	[GenBank:AY601633]	**53**	25	41	61,95	26	46	80,95
av-22	[GenBank:FJ404771]	**153**	7	18	33	14	26	45
av-23	[GenBank:DQ149621]	**801**	10	22	41,95	14	29	63,8
av-24	[GenBank:DQ149622]	**802**	10,05	22	37	16	31	63,95
av-25	[GenBank:DQ149623]	**808**	11	22	45			
av-26	[GenBank:EF153474]	**119**	8	18	36	14,05	27	58,95
av-27-02	[GenBank:DQ149625]	**65**	22,05	53	73			
av-27	[GenBank:AB330108]	**805**	12	23	48,95	16	34	66,7
av-28	[GenBank:FJ824826]	**151**	8	18	34,95	13	25	45,95
av-29-02	[GenBank:DQ149627]	**65**	22	52	75,9			
av-29	[GenBank:AB330110]	**802**	11	22	41,95	15	31	57,95
av-30	[GenBank:DQ149628]	**809**	9,05	23	48,95			
av-31-02	[GenBank:AB330111]	**63**	63	75	105	64	78	199,6
av-31	[GenBank:DQ149611]	**62**	22	45	68,95	28	50,5	97,7
av-32	[GenBank:DQ149629]	**833**	10	23	41,95			
av-33	[GenBank:DQ149630]	**803**	9,05	21	43,9	16	33	71,95
av-34	[GenBank:AY737797]	**230**	18	30	49	26	45	73,9
av-35	[GenBank:AC_000019] [GenBank:BK005236]	**125**	18	30	56,95	26,05	45	69
av-36	[GenBank:GQ384080]	**804**	9	23	46,95			
av-37	[GenBank:AB448778]	**123**	8	19	39	14	27	51,9
av-38	[GenBank:DQ149633]	**828**	10,05	23	49,95			
av-39	[GenBank:DQ149634]	**814**	11	22,5	45,85			
av-40-f	[GenBank:L19443]	**319**	13	27	56	19,05	33	65
av-41	[GenBank:HM565136]	**4136**	14	27	56,9	22	39	73,95
av-42	[GenBank:DQ149635]	**814**	11	24	43,9	20	37	82,95
av-43	[GenBank:DQ149636]	**811**	10,05	23	48,8			
av-44	[GenBank:DQ149637]	**802**	8	21	45,85			
av-45	[GenBank:DQ149638]	**821**	10	23	45,9			
av-46	[GenBank:AY875648]	**114**	8	18	34	14	26	49,95
av-47	[GenBank:DQ149640]	**807**	10,05	24	56			
av-48-merged	[GenBank:EF153473]	**149**	10	18	35,95	14	27	54
av-49-merged	[GenBank:DQ393829]	**217**	8	18	32,95	16	28	59,95
av-50-merged	[GenBank:AY737798]	**83**	12	28	47,9	21,05	38	59
av-51	[GenBank:DQ149642]	**828**	10,05	23,5	52,95			

herp1	[GenBank:NC_001806]	**2480**	8	17	33	13	26,5	54
herp2	[GenBank:NC_001798]	**79163**	10	20	36	19	35	63
herp3	[GenBank:NC_001348]					23	41	70
herp4 type1	[GenBank:NC_007605]	**1114**	23	38	64	29,05	47	63
herp4	[GenBank:NC_009334]	**516**	25	39	58,95	29	47	69,95
herp5	[GenBank:NC_006273]	**1215**	8	16	34,95	17	33	78
herp6a	[GenBank:NC_001664]	**15330**	56	68	99,9	68,05	118	2272
herp6b	[GenBank:NC_000898]	**15313**	56	68	114	1653	6483	13459
herp7	[GenBank:NC_001716]	**40093**	85	108	217	84929	1E+05	1E+05
herp8	[GenBank:NC_009333]	**2478**	13	24	46,85	16	29	51

Bacillus phage BCJA1c	[GenBank:NC_006557]	**75**	53	65	84	53,05	65	100,7
Burkholderia phage BcepNY3	[GenBank:NC_009604]	**17**	2	5	12,95	2	5	13
Clostridium phage phi CD119	[GenBank:NC_007917]	**344**	197	263	1028	194	247	454,5
Klebsiella phage KP32	[GenBank:NC_013647]	**20**	7	16	30	7	16	31,9
Listeria phage B025	[GenBank:NC_009812]	**11268**	84	105	173,8	83	106	171,8
Pseudomonas phage phi-2	[GenBank:NC_013638]	**58**	4,05	12	23,95	4	13	35,85
Staphylococcus phage 53	[GenBank:NC_007049]	**119**	76	95	150,8	76	93	135,9
Halomonas phage phiHAP-1	[GenBank:NC_010342]	**21**	3	9	21,95	2,05	9	22
Mycobacterium phage Cali	[GenBank:NC_011271]	**51**	3,05	10	24	5	12	22
Pseudomonas phage DMS3	[GenBank:NC_008717]	**12**	4	10	26,95	4	10	24,9
Pseudomonas phage SN	[GenBank:NC_011756]	**27**	9	19	33	9	20	38
Rhizobium phage 16-3	[GenBank:NC_011103]	**14**	4	12	25	5	13	26,95
Xylella phage Xfas53	[GenBank:NC_013599]	**52**	4	12	24	4	12	28,9

^1^Virus title. AV 01-51, adenoviral genomes (green). Herp 1-8, herpes virus genomes (yellow). The bacteriophagal genomes are shown in pink.

^2^The total number of BLAST hits found for the original viral genome. The default "Nucleotide BLAST" for the "BLAST at NCBI" search criteria were used.

^3^The data obtained for the sets of random genomes generated using Markov chain 2^nd ^- and 5^th ^order algorithm. 1.000 random sequences were generated for every existing viral genome. Expected numbers of BLAST hits for the 5^th^, 50^th ^and 95^th ^percentiles are given, accordingly. The data was generated using specially designed script. The script "BioVictor-Python1" is available upon the request to the authors.

**Figure 3:**
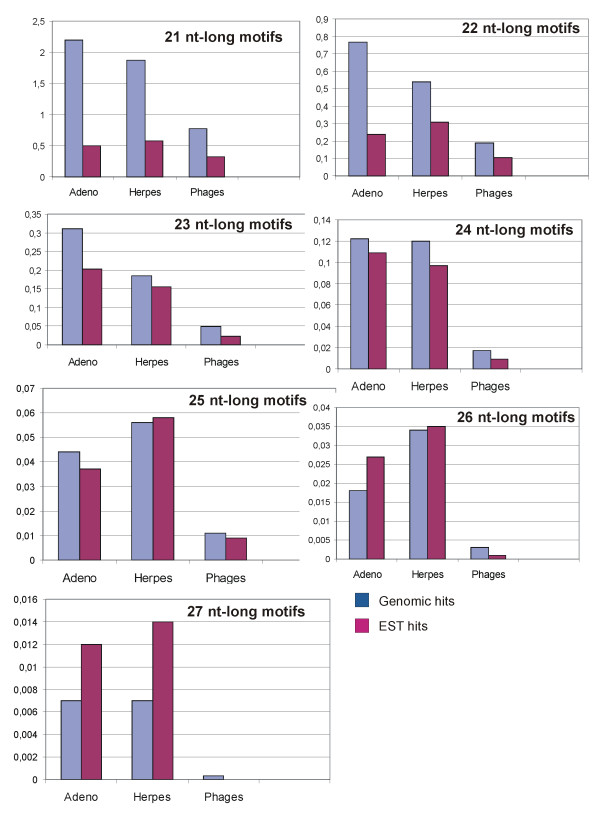
**Genomic and transcribed sequences identical between the human and viral genomes**. Human adenoviral, human herpes viral and bacteriophagal genomes were analyzed. Blue columns delineate BLAST hits found in human genomic DNA databases (non-redundant sequences + HTGS), lilac - hits identified using human expressed sequence tag (EST) database. Ordinate axis represents average number of hits per 1 kb of virus genome sequence for each group.

Our further studies revealed that many human-virus BLAST hits appeared to be transcribed in human, as learned from the analysis of human EST database (Figure 3). As before, numbers of transcribed hits for herpes- and adenoviral genomes were far greater than those for the phages. Notably, there was a clear-cut tendency towards a greater representation of longer herpes- and adenoviral hits in the human EST database, compared to the absence of relatively "long" transcribed hits for the phages (Figure 3). In several cases the number of "transcribed" hits was even higher than the number of hits matching human genomic DNA database (adenoviral genomes, 26 nt-long motifs). Detailed analysis of those sequences revealed that this was due to transcriptional processing features such as splicing and polyadenylation that increased variability of the transcribed part of human genome.

Therefore, the results of this pilot assay point to accumulation and functional relevance of the infectious-virus-like sequences of the human genome.

**Figure 4:**
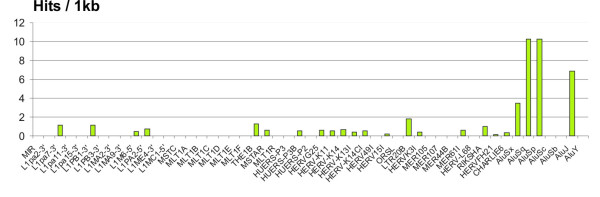
**Normalized content of the DNA motifs identical between the consensus sequences of human transposable elements and human herpesviral and adenoviral DNAs**. Bar heights are proportional to the relative content of perfectly matched BLAST hits per 1 kb of the respective TE group consensus sequence. Human TE consensus sequences were taken from the database RepbaseUpdate [45].

Many such identical motifs were parts of human transposable elements. For example, one 23 nt-long motif (*CGTACTTCAGCCTGGGCAACAAG*) that shared perfect sequence identity with three adenoviral genomes and considerable identities - with five other adenoviral genomes, was included in a variant of human transposable element of AluS family and was represented in the genome by multiple copies. We further investigated whether consensus sequences of other human TE families include sequence motifs perfectly matching to human adenovirus or human herpesvirus genomes. We were registering only the hits displaying perfectly matched 16 nt- long or more extended motifs (Figure 4). Such hits have been found for 23 out of 51 human TE families, and the distribution of hits there was not uniform. The highest relative numbers of identities per 1 kb of the TE consensus sequence were detected for different subfamilies of human retrotransposon Alu, which is known to be the most successful human TE in terms of propagation of its copies (over 1 million of copies per genome). Interestingly, it has been reported previously that adenoviral infection results in a dramatic increase in Alu transcription in human cells [40]. Our hypothesis might at least partly explain this phenomenon.

**Figure 5:**
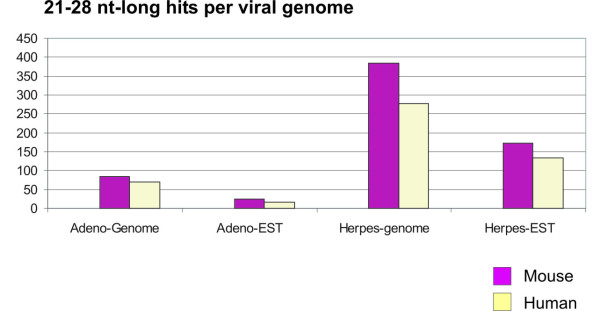
**Content of the mouse viral sequence motifs shared among the viral and mammalian DNAs**. Murine adenovirus A and murid herpesvirus 1 were screened against human and mouse genomic and EST databases. Bars delineate total numbers of 21-28 perfectly matched BLAST hits for each category.

We further screened available analogous mouse viral genomes (Murine adenovirus A and Murid herpesvirus 1) against human and mouse genomic and EST databases. For both mouse adenoviral and herpesviral genomes, the number of 21-28 nt-long hits was higher when searched through the mouse genomic and EST databases compared to the human ones (Figure 5).

Among the identified virus-like hits presented in both human and mouse DNAs, three sequences were simple repeats represented by multiple copies in both genomes (motifs *TGCTGATGCTGATGCTGATGCTGATG*, *CATCCATCCATCCATCCATCC *and *ATTCTTTCATTCTTTCATTCTTT*). Importantly, their copy numbers were very different in the mouse and human DNAs (mouse/human): 1216/194, 20384/13893 and 1120/192, respectively. Thus, a kind of positive selection for simple repetitive elements matching genomes of the viruses with the respective tropism may theoretically take place in this case.

Finally, in addition to antiviral adaptations the above identities of the host and viral genomes may also represent a virus adjustment to the host aimed at the regulation of the host gene expression that may facilitate viral life cycle progression (reviewed in [27]). Both lines of co-evolution are possible, and detailed experimental studies will be necessary to explore each case of the host-virus sequence coincidence.

## Testing the hypothesis

### Objects

Objects for testing this hypothesis could be various mammalian species and viruses infecting mammalian cells.

### Experiments

Apart from investigating susceptibility to viral infections, many other types of experiments can be proposed to test this hypothesis. For example, the comparisons of host and viral DNAs can be done in order to identify homologous nucleotide motifs. It can be further investigated (*e.g*., using Northern blot or microarray hybridization) whether there are such motifs transcribed in the antisense orientation relatively to viral gene transcriptional direction. For those transcribed in the antisense orientation relatively to viral gene expression, complete host RNA primary structures can be established (*e.g*., using 5'- and 3' RACE technique [41]). These RNAs may be assayed in functional tests whether they do interfere with viral gene expression and progression of ongoing infection using multiple *in vitro *and *in vivo *approaches (*e.g*., by assessing the effects of overproducing RNAs of interest on viral infection or viral gene expression).

## Implications of the hypothesis

It is proposed here that non protein-coding parts of the mammalian transcriptomes include thousands of nucleotide motifs that can be employed to suppress viral gene expression. We hypothesize that the evolutionary success of some families of mammalian transposable elements may at least partly be due to their ability to provide substantial amounts of antiviral RNAs.

It could be also generalized that theoretically species having increased genome sizes may resist various viral infections stronger than related organisms with more compact genomes. A practical implication probably might be that introducing artificially engineered TE sequences encoding antiviral RNAs could be advantageous for creating strains and breeds of eukaryotic organisms with the complex genomes that would be more resistant against intracellular parasites, e.g. for the needs of plant bioengineering. However, in this case enlargements of genomic DNAs must be followed by artificially accelerated mutation processes in order to increase genome diversity and, therefore, to create additional structure motifs potentially interfering with viral expression. The latter goal could be achieved using a wide number of available physical or molecular methods like gamma-irradiation and treatment with various mutagenic chemicals [42,43]. Moreover, quick evolution of the pathogenic viral genomes may be somewhat compensated by the accumulation of mutations in multiple TE copies which might significantly strengthen antiviral response.

## Conclusions

It is proposed that mammalian genomes contain thousands of relatively short sequence motifs that may be beneficial to the host organisms as a source of putative interfering RNA molecules that can be recruited to repress intracellular pathogens like viruses. We identified a large number of short sequences (21-29 bp long) in human genome that are identical to sequences of different types of human adenoviruses and herpesviruses. Many such motifs are transcribed and may be involved in RNAi-mediated defense to viral infection. In this case, RNAi could serve as an intracellular "immune system" by repressing transcription of intra-genomic parasites like active viruses. We hypothesize here that that the evolutionary success of some types of mammalian genomic repeats and, in particular, of some TE families may at least partly be due to their ability to provide substantial amounts of antiviral RNAs.

## Abbreviations

RNAi: RNA interference; siRNA: small interfering RNA(s); dsRNA: double stranded RNA(s); TE: transposable elements; RACE: rapid amplification of cDNA ends.

## Competing interests

The authors declare that they have no competing interests.

## Authors' contributions

AZ participated in the sequence alignment, in the recovery of the identical motifs between the mammalian and viral genomes, and took part in writing the paper. VT did the major part of sequence alignment and statistic analyses. FF participated in the hypothesis presentation and in writing the paper. AB formulated the hypothesis, conceived of the study, and participated in its design and coordination, and drafted the manuscript. All authors read and approved the final manuscript.

## Reviewer's comments

### Reviewer's report 1

Eugene V. Koonin (The National Center for Biotechnology Information, NLM, NIH, Bethesda, USA)

#### Reviewer comments

Zabolotneva and Buzdin speculate that animal genomes expand under selective pressure for generation of antiviral siRNAs. They illustrate the hypothesis by identifying multiple 20-25 bp sequences identical to sequences in adenovirus genome and also claim but do not show similar findings for herpesvirus genomes.

I have major comments on both the conceptual and technical levels. Conceptually, I am confident that the only answer the question posed in the title of the paper is:

No, and the question itself makes little sense. Enlargement of the genome cannot be a mechanism at any rate but, regardless of the semantics, to claim that it is an adaptation, even in the general sense, let alone specifically for antiviral defense, is an obvious fallacy. To be more specific, this idea assigns to the evolutionary the kind of foresight it can never possess. There is no good reason to question the population-genetic explanation of the major increase in genome size seen in vertebrates, namely, that the small effective population size of animals results in inefficient purifying selection and so provides fixation of even slightly deleterious features. The genome growth is a manifestation of this fundamental phenomenon and occurs by virtue of various processes the most important of which are random DNA duplication and propagation of transposable elements. It is a completely different matter than much (we currently do not have a clear idea just how much) of the junk DNA is co-opted for various functions including control of selfish elements, both transposons and viruses, which is indeed crucial.

The above is not an unqualified condemnation of this manuscript in its entirety. In principle, it could be salvaged by reformulating the hypothesis to "Are antiviral small interfering RNAs encoded in animal genomes?" When discussing this question, the authors should be clear about the major known mechanism of generation of antiviral siRNAs, namely, production from dsRNA through the action of the RISC complex.

#### Author's response

We agree. In the revised version, we re-formulated title of the manuscript which is now as follows: "How many antiviral small interfering RNAs may be encoded by the mammalian genomes?", and put numerous changes in text to avoid conceptual problems mentioned by the referee. Milestone references mentioned by Dr. Koonin were added to the manuscript and discussed in the text.

#### Reviewer comments

However, not all viruses produce dsRNA, and in any case, it would be quite interesting if animal genomes indeed encoded siRNA against viruses, in addition to known ones against transposable elements. In my view, to substantiate such a hypothesis, several types of analysis are necessary: (i) expand the analysis of virus-specific sequences (at least, include the herpesvirus data but better additional families of viruses), (ii) compare the occurrence of virus-specific sequences to the random expectation and calculate p-values, (iii) examine the available transcriptome data for the presence of these sequences in transcripts, (iv) investigate the distribution of these sequences in the genome - are they found primarily in introns or in intergenic regions or randomly? If these results of such analysis point to functional relevance, this could become a stimulating hypothesis.

#### Author's response

We are extremely grateful for these advices by the referee. In the revised version, we expand the analysis to the additional 10 human herpesviral and 13 different bacteriophagal genomes and statistically tested the data. We also compare complementary motif occurrences in viral DNA with four randomly generated 50 kb-long "genomes". Furthermore, we have analyzed distribution of the EST hits among the different viral entries and obtained the data that hopefully somewhat support our hypothesis.

### Reviewer's comments on the revised version

#### Reviewer comments

In the revised version of their manuscript, Zabolotneva and coworkers eliminated the major misconceptions of their original manuscript and added some computational analysis that aim at demonstrating the plausibility of their idea that large mammalian genomes encode numerous antiviral microRNAs. The removal of the "teleological" aspects of the original article certainly makes the new version more palatable, and an attempt to incorporate more detailed sequence analysis is in itself laudable. However, unfortunately, problems remain. The bioinformatic analysis included in the paper is not professional. The authors give no p-values for the excess of the virus-specific motifs that they discover and do not explain the method the use to generate their random sequences. Accordingly, all the analysis is out of context and has no real meaning as there is no indication whether or not the excess of hits in viral genomes compared to random sequences and phages is statistically significant or not.

#### Author's response

In the present version of the paper, the excess of the virus-specific motifs is shown to be statistically significant. The calculated p-values are given in the separate table (Table 1).

#### Reviewer comments

It would be advisable to calculate p-values both analytically and by comparison with random sequences that would have to be generated by shuffling the actual viral genomes (preferably, trying Markov models of different orders). Under this approach, it is necessary to generate many (at least, 1000, and preferably, more) random sequences separately for each viral genome and determine where in the distribution of the number of hits is the real genome. It also would be curious to reproduce this procedure with bacteriophage genomes (very strangely, in the current version, the authors do not specify which page genomes they used).

#### Author's response

In the new version, we specify adenoviral, herpesviral and phage genomes in the tables 2 and 3.

**Table 3:** List of viral genomes under investigation

Virus type	Genome size, kb	Accession number
Human adenovirus type 1	36.001	[GenBank:AC_000017] [GenBank:BK005234]

Human adenovirus type 2	35.937	[GenBank:AC_000007]

Human adenovirus type 3, strain GB	35.345	[GenBank:NC_011203]

Human adenovirus type 4, strain NHRC 3	35.964	[GenBank:AY599837]

Human adenovirus type 5	35.938	[GenBank:AC_000008]

Human adenovirus type 7	35.514	[GenBank:AC_000018] [GenBank:BK005235]

Human adenovirus type 8	34.980	[GenBank:AB448769]

Human adenovirus type 9	35.083	[GenBank:NC_010956]

Human adenovirus type 11	34.794	[GenBank:AC_000015] [GenBank:BK001453]

Human adenovirus type 12	34.125	[GenBank:NC_001460]

Human adenovirus type 14	34.763	[GenBank:FJ822614]

Human adenovirus type 16	35.552	[GenBank:AY601636]

Human adenovirus type 17	35.100	[GenBank:AF108105]

Human adenovirus type 19	35.231	[GenBank:AB448774]

Human adenovirus type 22, isolate AV-2711	35.166	[GenBank:FJ404771]

Human adenovirus type 26	35.152	[GenBank:EF153474]

Human adenovirus type 28, strain BP-5	35.130	[GenBank:FJ824826]

Human adenovirus type 34, strain Compton	34.755	[GenBank:AY737797]

Human adenovirus type 35	34.794	[GenBank:AC_000019] [GenBank:BK005236]

Human adenovirus type 37	35.152	[GenBank:AB448778]

Human adenovirus type F	34.214	[GenBank:L19443]

Human adenovirus type 41, isolate TAK	34.188	[GenBank:DQ315364]

Human adenovirus type 46	35.178	[GenBank:AY875648]

Human adenovirus type 48	35.206	[GenBank:EF153473]

Human adenovirus type 49	35.215	[GenBank:DQ393829]

Human adenovirus type 50, strain Wan	35.385	[GenBank:AY737798]

Murine adenovirus A	30.944	[GenBank:NC_000942]

Human herpesvirus 1	152.261	[GenBank:NC_001806]

Human herpesvirus 2	154.746	[GenBank:NC_001798]

Human herpesvirus 3	124.884	[GenBank:NC_001348]

Human herpesvirus 4	171.823	[GenBank:NC_007605]

Human herpesvirus 4, type 2	172.764	[GenBank:NC_009334]

Human herpesvirus 5	235.646	[GenBank:NC_009334]

Human herpesvirus 6	159.322	[GenBank:NC_009334]

Human herpesvirus 6, type 2	162.114	[GenBank:NC_000898]

Human herpesvirus 7	153.080	[GenBank:NC_001716]

Human herpesvirus 8	137.969	[GenBank:NC_009333]

Murid herpesvirus 1	230.278	[GenBank:NC_004065]

Microcystis phage MA-LMM01	169.109	[GenBank:NC_008562]

Bacillus phage 0305 phi 8-36	218.948	[GenBank:NC_009760]

Pseudomonas phage DMS 3	36.415	[GenBank:NC_008717]

Enterobacteria phage HK022	40.751	[GenBank:NC_002166]

Enterobacteria phage lambda	48.502	[GenBank:NC_001416]

Mycobacterium phage Cali	155.372	[GenBank:NC_011271]

Pseudomonas phage SN	66.390	[GenBank:NC_011756]

Rhizobium phage 16-3	60.195	[GenBank:NC_011103]

Burkholderia phage BcepNY3	47.382	[GenBank:NC_009604]

Klebsiella phage KP32	41.119	[GenBank:NC_013647]

Pseudomonas phage phi-2	43.144	[GenBank:NC_013638]

Halomonas phage phiHAP-1	39.245	[GenBank:NC_010342]

Xylella phage Xfas53	36.674	[GenBank:NC_013599]

Randomly generated genome 1	50.000	-

Randomly generated genome 2	50.000	-

Randomly generated genome 3	50.000	-

Randomly generated genome 4	50.000	-

As suggested by the referee, we compared human-virus sequence identities using a panel of randomly generated genomes. Using 2^nd ^- and 5^th ^order Markov model we generated random sequences by shuffling the actual adenoviral, herpesviral and bacteriophagal genomes. Under this approach, 1000 random sequences were generated separately for each investigated viral genome. We next compared numbers of BLAST hits for the existing viral genomes, and for *in silico *generated ones. The observed BLAST hits (total numbers of BLAST hits were found for each genome) were statistically analyzed (Table 2), and the following was found: (*i*) the number of hits for the existing adenoviral or herpesviral genome was mostly higher than the 95^th ^percentile for a set of the corresponding *in silico*-generated sequences (in 81% or 89% of the cases, respectively, for 5^th ^order Markov model), (*ii*) for the bacteriophages, this number was mostly below the 95% percentile and expanded it only in 38% of the cases). Again, these data confirm that human-pathogenic viruses share significantly greater structural identity with the human genome than do the bacteriophagal genomes. Importantly, this also implies that there was a kind of positive selection for either the "virus-like" sequences in the human DNA, or for the "humanized" DNA in the human-pathogenic viruses, or both.

#### Reviewer comments

Beyond these technical issues, the article still involves some conceptual vagueness. What is the authors' hypothesis on the origin of the putative antiviral RNA? Do they think that these sequences were acquired by insertion of virus-specific DNA or have they just emerged by chance? Both possibilities appear realistic, and the choice of the best interpretation, to a large extent, depends on the results of the statistical analysis outlined above. Regardless, it is highly desirable to be clear about the mechanistic aspects of the hypothesis.

#### Author's response

In the new version, we state that: "there was a kind of positive selection for either the "virus-like" sequences in the human DNA, or for the "humanized" DNA in the human-pathogenic viruses, or both." At present, we cannot be more certain about what flow in human-virus DNA interchange is the most important.

#### Reviewer comments

The language of the manuscript remains quite poor. The text might have been seen by a professional translator who might have removed some of the errors but many of these remain along with the overall poor style.

#### Author's response

Native English-speaking colleague edited the text.

### Reviewer's report 2

Valerian V. Dolja (Department of Botany and Plant Pathology, Oregon State University, Corvallis, USA)

#### Reviewer comments

This Hypothesis article by Anastasia Zabolotneva and Anton Buzdin advances a concept according to which the eukaryotes with large (e.g., polyploid) genomes take advantage of a large supply of genetic material that is not a subject of purifying selection to evolve antiviral RNA transcripts. It is further proposed that such transcripts could activate RNAi machinery and therefore suppress the infection.

This concept is well in line with the recent striking findings of the bacterial anti-phage CRISPR defense system and growing number of instances of acquisition of RNA virus sequences by eukaryotic genomes that were also proposed to function in antiviral defense (see a succinct commentary by Eugene Koonin, 2010). Although a welcome generalization, current Hypothesis appears to be rather thin on supporting evidence and lacking in specifics as it comes to the involved molecular mechanisms. Below is a laundry list of comments addressing which could, in my opinion, strengthen the case made by the authors.

1. The only bioinformatics support for the hypothesis comes from finding a substantial number of short sequences identical to human adenoviruses in the human genome. It is not clear, however, if there is any positive selection/enrichment for such sequences or, if their occurrence is purely incidental. It seems that a simple *in silico *experiment could provide an important insight into this issue. If the genomes of DNA phages similar in size to adenoviruses are used as a query, will there be a similar or significantly lower number of hits? Since phages do not infect humans, the latter outcome would be supportive of positive selection for the retention of human virus related sequences rather than for mere stochastic occurrence of the irrelevant sequences.

#### Author's response

In the revised version - done exactly as suggested by the referee (see also our reply #2 to the reviewer 1).

#### Reviewer comments

2. Along the similar lines, it is not specified if there are any pathways in addition to positive selection (that in itself could be insufficient) that allows for selective retention of antiviral sequences as opposed to those affecting human own genes. Again, a simple search for, e.g., sequences identical to ribosomal RNAs (outside the rRNA genes proper) could provide relevant insight.

#### Author's response

We omitted this type of analysis suggested by the referee because mammalian genomes (and human genome as well) contain huge numbers of pseudogenes for rRNA that significantly bias interpretation of the data.

#### Reviewer comments

3. Proposed hypothesis testing via comparing viral susceptibility of the closely related organisms with contrasting genome size appears to be conceptually problematic. The case in point is plant species that underwent evolutionary recent polyploidy transitions. Such plants tend to be more rapidly growing and vigorous than their diploid kin. Consequently, if the former are found to be more virus-resistant than the latter, it could be attributed to their overall vigor (and/or increased complement of innate and acquired immunity genes) rather than to acquisition of additional antiviral sequences.

#### Author's response

We agree. The paper has been seriously revised and the confusing part was removed from the manuscript.

#### Reviewer comments

4. It is not clear why the RNAi-based antiviral response invoked in the paper is habitually called 'intercellular' immune system. Even though cell-to-cell and long-distance spread of RNAi signaling is described for plants and C. elegans, by and large, the RNAi machinery is cell-autonomous, that is, is expressed in each cell.

#### Author's response

Corrected through the manuscript.

#### Reviewer comments

The paper needs to be heavily edited against numerous typos (e.g. 'specie' throughout the text), as well as grammatical and stylistic errors (e.g., 'Theoretically, a practical implication could be...', on p. 8; an oxymoron).

#### Author's response

A professional interpreter edited the revised version.

### Reviewer's report 3

Yuri V. Shpakovski (Shemyakin-Ovchinnikov Institute of Bioorganic Chemistry, Russian Academy of Sciences, Moscow, Russia)

#### Reviewer comments

I was hesitant to review the original version of the manuscript because the general idea of the paper in its original form (Zabolotneva A. & Buzdin A.: Hypothesis: may enlargement of eukaryotic genome size be a mechanism of anti-viral host cell defense?) was really misleading - in my opinion the case presented have merely nothing to do with the enlargement of eukaryotic genomes and with the so-called C-value or G-value paradoxes. Unfortunately, the remnants of this misconception are still in the paper's text (e.g. the idea of creating "polyploid eukaryotic organisms that would be more resistant against intracellular parasites ... for the needs of plant bioengineering"), and without this strange 'genome size association' (which I believe to be wrong) the so-called hypothesis presented in the manuscript ("We propose a hypothesis that eukaryotic genomes encode short RNA motifs that may serve for the antiviral protection...") does not have so much actual novelty.

Indeed, a very nice review of Gottwein E. & Cullen B.R. "Viral and cellular microRNAs as determinants of viral pathogenesis and immunity" (published in Cell Host Microbe, 2008, Vol. 3, No. 6, pp. 375-387) not only contains more wide, stimulating and scientifically grounded discussion of the involvement of RNAi mechanisms in virus-host interactions, but even offers some experimentally verified examples of the impact of host miRNAs on viral replication and pathogenesis.

Authors of the new submission must clearly and unequivocally specify what real novelty their hypothesis contains: the general answer on the question posted in a new title (Are antiviral small interfering RNAs encoded in EUKARYOTIC genomes?) after such for some reason omitted in the paper's Reference list publications as Lecellier et al. (Science, 2005, **308**: 557-560), Otsuka et al. (Immunity, 2007, **27**: 123-134), Pedersen et al. (Nature, 2007, **449**: 919-922), Ahluwalia et al. (Retrovirology, 2008, **5**:117) is obvious YES. All these publications show that some cellular miRNAs play a role in direct or indirect regulation of viral genes. These small interfering RNAs are broadly implicated in viral infection of mammalian cells, with either positive or negative effects on viruses' replication. And I am talking here only about mammalian species, because the involvement of RNAi in the innate antiviral immune response in plants and invertebrate animals is already well documented.

#### Author's response

We are very thankful to the referee for his valuable criticism. Indeed, the initial version of the manuscript was greatly overlapping with the abovementioned papers. In the present version, an attempt has been made to avoid ambiguous sentences, e.g. concerning polyploid organisms. As to the novelty, to meet the referee suggestion we revised the major concept of the manuscript. We propose a hypothesis that mammalian DNAs and, in particular, human genome, encode thousands of the RNA motifs that may serve for the antiviral protection. We also presume that the evolutional success of some groups of genomic repeats and, in particular, transposable elements (TEs) may be due to their ability to provide to the host organism antiviral RNA motifs. Genomic repeat intense propagation into the genome inevitably causes bidirectional transcription of these sequences, and the resulting double-stranded RNAs may be recognized and processed by the RNA interference enzymatic machinery. Provided that these processed target motifs may be complementary to viral transcripts, fixation of the repeats into the genome may be of a considerable benefit to the host.

#### Reviewer comments

I also have some comments concerning the computational data presented in a new manuscript's submission. The comparison of the occurrence of virus-specific sequences to the random expectation or to bacteriophagal sequences has a very limited scientific value (if any) - of course, the genomes interacting in the course of evolution have more in common than evolutionarily unrelated or artificially chosen sequences. More relevant to the case presented could be testing by bioinformatics means the virus-host specificity of the discussed short RNA motifs present in different mammalian species: was there any co-evolution of the viral and host-acquired sequences or not?.. The positive correlation could probably strengthen the case. Particularly, this kind of viral-host sequence comparison could be done using as queries genomes of the viruses for which is already known that they are using RNAi machinery in their interaction with the hosts: human and mouse cytomegaloviruses (hCMV and mCMV), human, simian and murine rhadinoviruses (KSHV, RRV, MHV68), human and rhesus lymphocryptoviruses (EBV & rLCV).

#### Author's response

We added the results of some additional bioinformatical tests to the present version. We extracted from genomic databases the available mouse adenovirus and herpesvirus genomes (Murine adenovirus A and Murid herpesvirus 1) and screened them against human and mouse genomic and EST databases. For both mouse adenoviral and herpesviral genomes, the number of 21-28 nt-long hits was higher when searched through the mouse genomic and EST databases compared to the human databases. Among the identified virus-like hits presented in both human and mouse DNAs, three sequences were simple repeats represented by multiple copies in both genomes (motifs *TGCTGATGCTGATGCTGATGCTGATG*, *CATCCATCCATCCATCCATCC *and *ATTCTTTCATTCTTTCATTCTTT*). Importantly, their copy numbers were very different in the mouse and human DNAs (mouse/human): 1216/194, 20384/13893 and 1120/192, respectively. Thus, a kind of positive selection for simple repetitive elements matching genomes of the viruses with the respective tropism may theoretically take place in this case. Overall, these data are supportive towards the general concept of this manuscript. We have also tested the presence of adeno- and herpesvirus-like motifs in the consensus sequences of human transposable elements and found that abundant genomic Alu repeats are enriched in such elements. We thank the referee for recommending a strategy of further studies that would include subsequent comparisons of various herpesviral genomes with the DNAs of their hosts and vice versa. However, these studies go beyond the scope of this hypothesis paper and will be a matter of our further research projects that would include also a detailed analysis of coevolution of genomic repeats, viruses and their hosts for various mammalian organisms.
